# Breast microbiome associations with breast tumor characteristics and neoadjuvant chemotherapy: A case-control study

**DOI:** 10.3389/fonc.2022.926920

**Published:** 2022-09-12

**Authors:** Xuejun Li, Xiaohu Sun, Ai Zhang, Jing Pang, Yun Li, Mengfan Yan, Zhen Xu, Yue Yu, Zhengjun Yang, Xi Chen, Xin Wang, Xu-Chen Cao, Nai-jun Tang

**Affiliations:** ^1^ Department of Occupational and Environmental Health, School of Public Health, Tianjin Medical University, Tianjin, China; ^2^ Tianjin Key Laboratory of Environment, Nutrition and Public Health, Tianjin, China; ^3^ Center for International Collaborative Research on Environment, Nutrition and Public Health, Tianjin, China; ^4^ The First Department of Breast Cancer, Tianjin Medical University Cancer Institute and Hospital, National Clinical Research Center for Cancer, Tianjin, China; ^5^ Key Laboratory of Breast Cancer Prevention and Therapy, Tianjin Medical University, Ministry of Education, Tianjin, China

**Keywords:** Breast cancer, microbiome, normal adipose tissue, neoadjuvant chemotherapy, 16S rRNA gene sequencing

## Abstract

**Background:**

Commensal microbiota have been proven to colonize the mammary gland, but whether their composition is altered in patients with breast cancer (BC) remains elusive. This study intends to explore the breast microbiome differences between benign and malignant diseases and to investigate the impact of neoadjuvant chemotherapy (NAC) on the breast microbiota in patients with BC.

**Methods:**

Breast normal adipose tissues (NATs) were collected from 79 patients with BC and 15 controls between July 2019 and November 2021. The BC group consisted of 29 patients who had received NAC and 50 who were non-NAC patients. Participants diagnosed with benign breast disease were recruited as controls. 16S rRNA gene sequencing was used to analyze the bacterial diversity of NATs.

**Results:**

The community structure of the NAT microbiome was significantly different between the BC and control groups. *Proteobacteria* decreased (47.40% versus 39.74%), whereas *Firmicutes* increased (15.71% versus 25.33%) in patients with BC when compared with that in control tissues. Nine genera were enriched in BC NATs, and four genera levels increased in the control group. The associations between differential bacterial genera and breast tumor grade were calculated by Spearman’s correlation. The results showed that tumor grade was positively associated with the relative abundance of *Streptococcus* and negatively related to *Vibrio*, *Pseudoalteromonas*, *RB41*, and *Photobacterium*. Moreover, menopause was associated with the microbiota composition change of non-NAC BC patients and related to the significant reduction in the abundance level of *Pseudoalteromonas*, *Veillonella*, and *Alcaligenes*. In addition, NAC was related to the beta diversity of patients with BC and associated with the decrease of *Clostridium_sensu_stricto_7* and *Clostridium_sensu_stricto_2* in postmenopausal patients. Of note, Tax4Fun functional prediction analysis revealed that the metabolic state was more exuberant in the BC group with upregulating of multiple metabolism-related pathways.

**Conclusion:**

Our results offer new insight into the relationship between NAC and breast microbiota and help to better characterize the breast microbial dysbiosis that occurs in patients with BC. Further epidemiological studies with larger sample size and well-designed animal experiments are required to elucidate the role of breast microbiota in the therapeutic outcome of BC.

## Introduction

Breast cancer (BC), with 2.3 million new cases (11.7%) in 2020, is the most common carcinoma worldwide. Death caused by BC ranked fifth in the global cancer statistics in 2020 (1). Such staggering numbers still exist despite breakthrough advancements in diagnosis and treatment. The precise etiology of BC and novel approaches for targeting this type of cancer need in-depth research in the future to cause a decline in BC-induced mortality rates (2). BC is a multifactorial disease that has been attributed to a series of important risk factors, including genetic predisposition, exposure to estrogens (endogenous and exogenous), low parity, high breast density, and a history of atypical hyperplasia (3). Of note, the human microbiome is considered a new risk factor for BC and has attracted a lot of attention in recent years. The metagenome of the human microbiome is 100-fold more diverse compared with the human genome, which is critically associated with human health (4, 5). Breast tissue was initially considered to be a sterile tissue with no microbial population. The existence of microbes in breast tissue was first demonstrated in 2014 (6), and many subsequent studies have revealed the presence of a distinct local microbiota in the breast (7–9). Nevertheless, published studies (6, 10–13) that have specifically investigated the differences in breast tissue microbiota between patients with BC and controls are controversial. The mechanism remains unclear, and questions still exist as to what role the breast microbiome plays in BC. Therefore, more studies to explore the role of the microbiota in BC are needed.

As a common treatment of cancer, neoadjuvant chemotherapy (NAC) is usually implemented to treat patients with locally advanced BC with the aim of reducing tumor size and allowing more breast conservative therapy to be performed (14). The therapeutic effect of NAC varies significantly in term of some factors, such as expression of hormone receptors and menopausal status (15, 16). A clinical study showed that antibiotic administration leads to a reduction in NAC efficacy and was associated with worse BC prognosis (17). Nevertheless, the relationship between NAC and the change in breast microbiota is not well understood to date. The study conducted by Chiba et al. demonstrated that NAC cause a change in the microbiome of breast tumors and the genera *Brevundimonas* and *Staphylococcus* correlated with BC recurrence (18). Further analysis was not performed for the small sample sizes.

The chief characteristics of the malignant phenotype are heavily dependent on the interaction between cancer cells and their microenvironment (19). Adipocytes account for the largest proportion among the cells that comprise breast tissue and are considered to play a critical role in the tumor microenvironment of BC (20). Therefore, we collected the breast normal adipose tissues (NATs) from patients with BC and participants of benign breast disease (controls) and analyzed the differences between groups by 16S rRNA sequencing to explore the effect of adipose microenvironment on BC from the perspective of microbiome.

The intent of this study was two-fold: investigate the differences in breast microbiota between benign and malignant diseases and explore the impact of NAC on the microbiome of NATs in patients with BC.

## Materials and methods

### Study subjects

This study was conducted at the Tianjin Medical University Cancer Institute and Hospital (Tianjin, China) and approved by the Ethics Board of the Tianjin Medical University Cancer Institute and Hospital (approved protocol number: bc2021013). We recruited 79 BC cases diagnosed by pathological examination of tumor tissues. Twenty-nine patients receiving NAC before surgery, 50 patients without a history of NAC (non-NAC), and 15 controls with a history of benign breast disease (breast nodule) but whose tissues were free of cancer cells between July 2019 and November 2021 were enrolled in the study. Informed consent was obtained from all participants. Demographic and clinicopathological data were collected from the hospital’s electronic medical records.

### Biological samples

When subjects signed the informed consent, NATs were taken 3–5 cm apart from the breast tumor area during surgery and washed with sterile saline (0.9% NaCl) solution. Samples were rapidly transported to the laboratory and stored at −80°C until DNA extraction.

### Genomic DNA extraction and quantification

NAT homogenates pre-treated with Pathogen Lysis Tubes L (QIAGEN, Germany). Sample fluids (400 μl) were mixed with 100 μl of buffer ATL (QIAGEN, Germany) and then transferred to a fresh lysis tube. The lysis tube was placed on a bead mill homogenizer (Retsch, Germany) at a rate of 30 frequency/s for 30 min. Bacteria DNA was extracted using the IndiSpin^®^ Pathogen Kit (INDICAL BIOSCIENCE, Germany) following the manufacturer’s directions. NanoDrop2000c (Thermo Fisher Scientific, USA) was used to determine DNA concentrations. The purity of genomic DNA was detected by agarose gel electrophoresis, and DNA was diluted to 1 ng/μl with sterile water in centrifuge tubes.

### Bacterial 16S rRNA amplification, purification, library construction, and sequencing

The Phusion^®^ High-Fidelity PCR Master Mix with GC Buffer (New England Biolabs, USA) was used for 16S rRNA amplification. 16S rRNA genes were amplified using the specific barcoded primers of 806R for V4 hypervariable regions. The PCR products were detected by electrophoresis using 2% agarose gel. The target band was extracted or cleaned using the QIAquick Gel Extraction Kit (QIAGEN, Germany). Finally, the TruSeq^®^ DNA PCR-Free High-Throughput Library Prep Kit (Illumina, USA) was used for library construction, and quantification of a library was done using Qubit and real-time PCR. After the library was qualified, all samples were sequenced on the Illumina NovaSeq 6000 platform.

### Statistical analysis

Filtered sequences were clustered by 97% identity into operational taxonomic units (OTUs) using UPARSE (21), and the sequence of highest occurrence frequency in OTUs was regarded as the representative sequence. The Mother method and SSUrRNA database (22) of SILVA138 (23) were used to conduct species annotation analysis on OTU sequences (the threshold value: 0.8–1.0), and the community composition of each sample was determined at the taxonomic level (*kingdom*, *phylum*, *class*, *order*, *family*, *genus*, and *species*). The phylogenetic relationship of all representative sequences was obtained by MUSCLE (version 3.8.31) (24). Finally, the data of each sample were normalized on the basis of the smallest amount of data in the sample.

QIIME (version 1.9.1) was used to calculate the index of Observed Species and Shannon. The rank abundance curve and diagrams of principal coordinate analysis (PCoA) were drawn with R (version 2.15.3) using the statistical packages “ade4”, “ggplot2”, “WGCNA”, “stats”, and “vegan.” Linear discriminant analysis (LDA) effect size (LEfSe) was used for LEfSe analysis, and the default filter value of the LDA score was 3.5.

All statistical analyses were conducted using R (version 2.15.3). A two-sided test and *P*-value < 0.05 were considered statistically significant.

## Results

### Participant demographics and NAT characteristics

Details of the clinical characteristics of 94 participants are shown in **Table 1**. We enrolled 79 patients with BC and 15 controls. The control group was older than patients with BC (mean: 53.33 versus 52.90 years old; *p* < 0.05). The BC group consisted of 29 patients who had received NAC and 50 who were non-NAC patients. In addition, we collected clinical data concerning hormone receptor status and tumor histological grade of breast tumors in patients with BC (**Table 2**).

**Table 1:** Clinical characteristics of the patients with breast cancer and controls.

Variables		Breast cancer (n = 79)	Control (n = 15)	*P*-value
Age (years)		52.90 ± 8.69	53.33 ± 6.79	0.00
Age of menarche(years)		14.70 ± 1.62	13.53 ± 2.61	0.03
Menopausal status (%)	Premenopausal	35 (44.30)	8 (53.30)	0.52
	Postmenopausal	44 (55.70)	7 (46.70)	
Parity (%)	≤1	59 (74.70)	11 (73.30)	0.91
	≥2	20 (25.30)	4 (26.70)	

**Table 2:** Clinical characteristics of the neoadjuvant chemotherapy (NAC) and non-NAC patients.

Variables		Non-NAC (n = 50)	NAC (n = 29)	Total (n = 79)
Age (years)		54.36 ± 8.48	50.38 ± 8.62	52.90 ± 8.69
Age of menarche(years)		14.94 ± 1.60	14.28 ± 1.60	14.70 ± 1.62
Menopausal status (%)	Premenopausal	22 (44.00)	13 (44.83)	35 (44.30)
	Postmenopausal	28 (56.00)	16 (55.17)	44 (55.70)
Status of hormone receptor of breast tumors (%)	ER+	30 (60.00)	19 (65.52)	49 (62.03)
	ER−	20 (40.00)	10 (34.48)	30 (37.97)
	HER2+	20 (40.00)	10 (34.48)	30 (37.97)
	HER2−	30 (60.00)	19 (65.52)	49 (62.03)
	Non-TNBC	40 (80.00)	24 (82.76)	64 (81.01)
	TNBC	10 (20.00)	5 (17.24)	15 (18.99)
Tumor grade (%)	Grade 1/2	28 (56.00)	18 (62.07)	46 (58.23)
	Grade 3	20 (40.00)	9 (31.03)	29 (36.71)
	Unknown	2 (4.00)	2 (6.90)	4 (5.06)

### Differences in NAT microbiome between patients with BC and controls

To avoid any bacterial contaminants that could occur during DNA isolation of the NAT samples, we extracted three cell line precipitates (MCF7, MDA-MB-231, and MCF-10A) as negative controls under the same experimental condition (**Supplemental Figure 1**). We used the 16S rRNA gene sequencing method to evaluate the absolute quantification of total bacterial load of NAT and cell line samples (**Supplemental Figure 1A**). The results revealed that the bacterial abundance was significantly greater in NATs than in cell lines (**Supplemental Figure 1B**).

**Figure 1:**
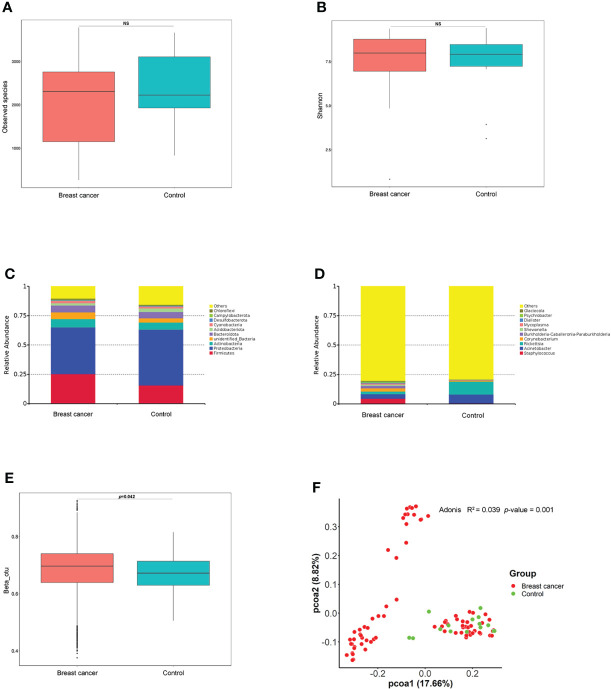
Differences in breast normal adipose tissue (NATs) microbiome between patients with breast cancer and controls. Alpha diversity was assessed by the Observed Species **(A)** and Shannon index **(B)**. The relative abundance of the taxonomic composition of the NATs microbiome at the phylum **(C)** and genus **(D)** levels. The differences in beta diversity were calculated by the Wilcoxon signed-rank test **(E)** and principal component analysis (PCoA) **(F)** of unweighted UniFrac distances (ns, *P*-value > 0.05).

The alpha diversity as assessed by the Observed Species (*p* = 0.22) and the Shannon index (*p* = 0.67) was similar between patients with BC and controls (**Figures 1A, B** **)**. *Proteobacteria* was the most abundant phylum in the total NATs, followed by *Firmicutes* and *Actinobacteria* (**Figure 1C**). Moreover, *Proteobacteria* decreased (47.40% versus 39.74%), whereas *Firmicutes* increased (15.71% versus 25.33%) in the BC group when compared with that in controls. At the genus level, *Staphylococcus* and *Acinetobacter* were the most abundant (**Figure 1D**) in the patients with BC, and *Rickettsia* and *Acinetobacter* were the most plenteous (**Figure 1D**) in the control group. The Wilcoxon signed-rank test and PCoA of unweighted UniFrac distances were used to investigate the differences in beta diversity between the BC and control groups. A comparison of NATs from patients with BC and controls showed distinctly different compositions of microbiota (*p* = 0.04) as shown in **Figure 1E**. The result of the PCoA of unweighted UniFrac distances was shown in **Figure 1F**. There were significant differences between the patients with BC and controls (Adonis, *p* = 0.001, R^2^ = 0.039). These findings suggest that breast microbiota dysbiosis may occur in patients with BC.

### Abundant microbes were significantly different between the BC and control groups

**Figure 2:**
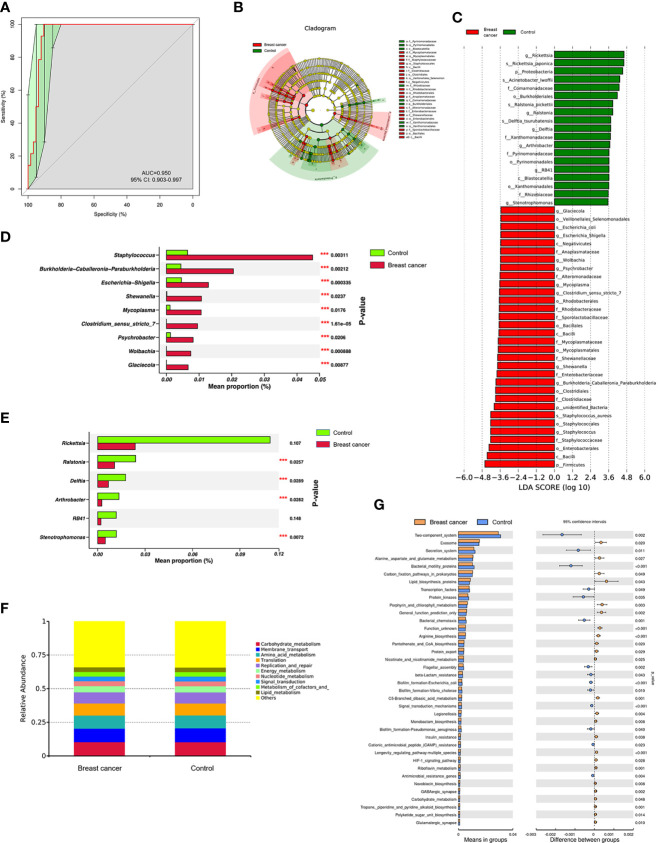
The abundant microbes significantly differed between the breast cancer (BC) and control groups. **(A)** The receiver operating characteristic (ROC) curve at the genus level. **(B, C)** The linear discriminant analysis (LDA) effect size (LEfSe) between the BC and control groups (LDA score > 3.5, *P*-value < 0.05). **(D, E)** The microbial differences at the genus level between patients with BC and controls (***P-value < 0.05). Tax4fun shows the different functional compositions of the breast normal adipose tissue (NAT) microbiome between the BC and control groups based on the Kyoto Encyclopedia of Genes and Genomes (KEGG) at level 2 **(F)** and level 3 **(G)**.

The receiver operating characteristic (ROC) curve was drawn at the level of genus between the BC and control groups. The area under the ROC curve (AUC) was 0.950, and the 95% confidence interval (CI) was 0.903–0.997 (**Figure 2A**), indicating that the differences in microbiome composition between the two groups occurred at the genus level. LEfSe and Wilcoxon signed-rank test analysis were further used to assess the differential genera between the BC and control groups. LEfSe analysis showed that significant differences in microbial abundance were observed between the two groups (**Figures 2B, C** **)**. Nine genera were overexpressed in patients with BC, and six genera were overrepresented in the control group (**Figure 2C**). Of note, the abundance levels of *Staphylococcus*, *Burkholderia-Caballeronia-Paraburkholderia*, *Escherichia-Shigella*, *Shewanella*, *Mycoplasma*, *Clostridium_sensu_stricto_7*, *Psychrobacter*, *Wolbachia*, and *Glaciecola* were elevated in the BC group (**Figure 2D**), and the levels of *Ralstonia*, *Delftia*, *Arthrobacter*, and *Stenotrophomonas* increased in controls (**Figure 2E**).

We used Tax4Fun to conduct functional prediction analysis (25) and predict the different functional compositions of the NAT microbiome between the BC and control groups based on 16S rRNA sequencing results. The top 10 common Kyoto Encyclopedia of Genes and Genomes (KEGG) categories at level 2 in the two groups were mainly related to metabolism (6/10, 60%) as shown in **Figure 2F**. Furthermore, we analyzed the differential categories based on the KEGG level 3 classification, and the results showed that six categories involved in metabolism, including “*Alanine, aspartate, and glutamate metabolism*”, “*porphyrin and chlorophyll metabolism*”, “*nicotinate and nicotinamide metabolism*”, “*C5-branched dibasic acid metabolism*”, “*riboflavin metabolism*”, and “c*arbohydrate metabolism*”, had significantly increased in the BC group (*p* < 0.05) as shown in **Figure 2G**, indicating that metabolic state was more dominant in the BC group compared with the controls.

### Multiple bacterial genera varied by tumor characteristics

**Figure 3:**
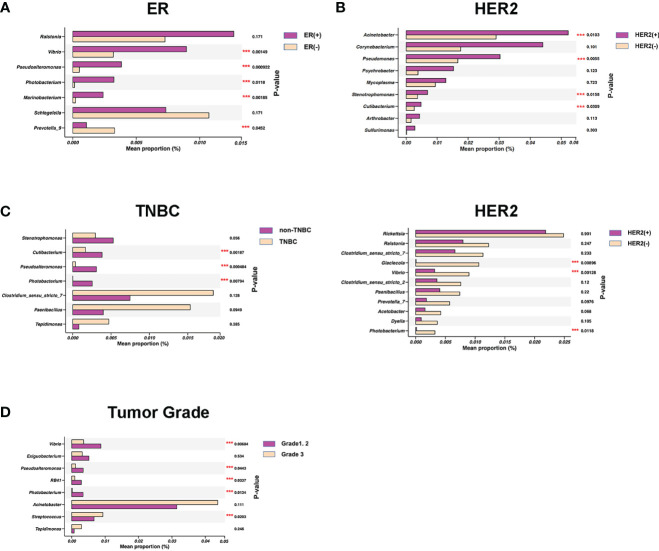
Specific bacterial genera in breast normal adipose tissues (NATs) correlated with the breast tumor characteristics. Mean relative abundance (proportions) of several bacterial genera were shown distinctively in estrogen receptor (ER)–positive versus negative **(A)**, human epidermal growth factor 2 (HER2)–positive versus negative **(B1, B2)**, non–triple-negative breast cancer (non-TNBC) versus TNBC **(C)**, and histological grades 1 and 2 versus grade 3 breast tumors **(D)** (***, *P*-value < 0.05).

Upon stratifying samples by the hormone receptor status of breast tumor, we noted that estrogen receptor (ER)–positive tumor NATs had higher abundance of four genera (*Vibrio*, *Pseudoalteromonas*, *Photobacterium*, and *Marinobacterium*) and lower abundance of *Prevotella_9* compared with ER-negative samples (**Figure 3A**). In comparison, human epidermal growth factor 2 (HER2)–positive tumor NATs had significantly higher abundance of four genera (*Acinetobacter*, *Pseudomonas*, *Stenotrophomonas*, and *Cutibacterium*) as shown in **Figure 3B1** and lower abundance of three genera (*Glaciecola*, *Vibrio*, and *Photobacterium*) compared with HER2-negative tissues (**Figure 3B2**). Meanwhile, three genera (*Cutibacterium*, *Pseudoalteromonas*, and *Photobacterium*) were relatively decreased in TNBC NATs compared with non-TNBC samples (**Figure 3C**).

Of note, we also identified that the NAT microbial markers were associated with tumor Nottingham grade. The tumor grade was positively associated with the relative abundance of *Streptococcus* and negatively related to four genera (*Vibrio*, *Pseudoalteromonas*, *RB41*, and *Photobacterium*) in NATs (**Figure 3D** and **Supplemental Table 1**). Collectively, these findings of both shared and distinct microbiota profiles associated with breast tumor characteristics revealed that the breast interactions between microbiome and tumor were complicated, which may involve in multiple factors.

### Microbial differences existed between the non-NAC and NAC patients

**Figure 4:**
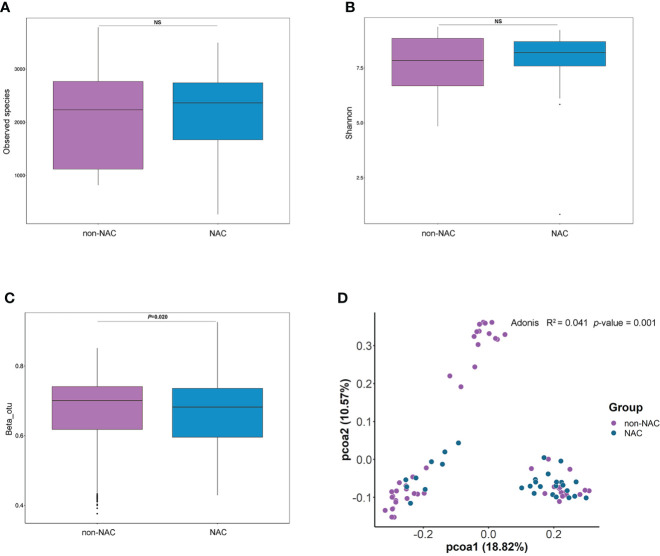
The differences in breast normal adipose tissue (NATs) microbiome between non-neoadjuvant chemotherapy (non-NAC) and NAC patients. The alpha diversity was assessed by the Observed Species **(A)** and Shannon index **(B)**. The differences in beta diversity were calculated by the Wilcoxon signed-rank test **(C)** and principal component analysis (PCoA) **(D)** of unweighted UniFrac distances (ns, *P*-value > 0.05).

Different indices (Observed Species and Shannon) were applied to evaluate the alpha diversity of the microbiome, and the results indicated that there were no significant differences between non-NAC and NAC patients (**Figures 4A, B** **)**. The comparison of NATs from the two groups showed distinctly different compositions of microbiota (*p* = 0.02) (**Figure 4C**). The results of PCoA and Adonis analyses indicated that a difference in microbiota structure existed between the non-NAC and NAC patients (**Figure 4D**). These findings suggested that the NAC may impact the balance of breast microbiome in patients with BC.

**Figure 5:**
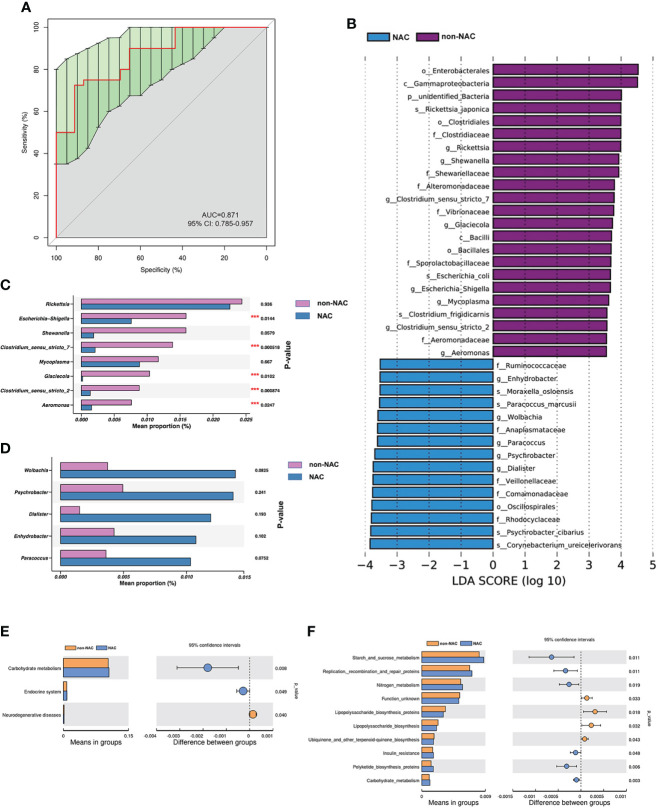
The abundant microbes significantly differed between the non-neoadjuvant chemotherapy (non-NAC) and NAC groups. **(A)** The receiver operating characteristic (ROC) curve at the level of genus. **(B)** The linear discriminant analysis (LDA) effect size (LEfSe) between non-NAC and NAC groups (LDA score > 3.5, *P*-value < 0.05). **(C, D)** The microbial differences at the genus level between non-NAC and NAC patients (***P-value < 0.05). Tax4Fun shows the different functional compositions of the breast normal adipose tissue (NAT) microbiome between the non-NAC and NAC groups based on the Kyoto Encyclopedia of Genes and Genomes (KEGG) at level 2 **(E)** and level 3 **(F)**.

The AUC was 0.871 (95% CI: 0.785–0.957) as shown in **Figure 5A**, indicating that the differences in microbiome composition between the non-NAC and NAC groups occurred at the genus level. The distinctions in taxa from phylum to genus levels were identified *via* the LEfSe analysis (**Figure 5B**). On the basis of the LEfSe findings at the genus level, we further assessed the bacterial biomarkers. The abundance levels of five genera (*Escherichia-Shigella*, *Clostridium_sensu_stricto_7*, *Glaciecola*, *Clostridium_sensu_stricto_2*, and *Aeromonas*) decreased when patients with BC received NAC (**Figures 5C, D** **)**. The differential categories between the non-NAC and NAC groups were related to *Carbohydrate metabolism* (**Figure 5E**). Of note, the results based on the KEGG level 3 classification calculated by Tax4Fun showed that three categories involved in metabolism both significantly increased in the NAC group compared with the non-NAC patients (*p* < 0.05) as shown in **Figure 5F**, indicating that NAC promoted the metabolic activities in patients with BC.

### The bacterial genera varied by menopausal status in the non-NAC and NAC patients

**Figure 6:**
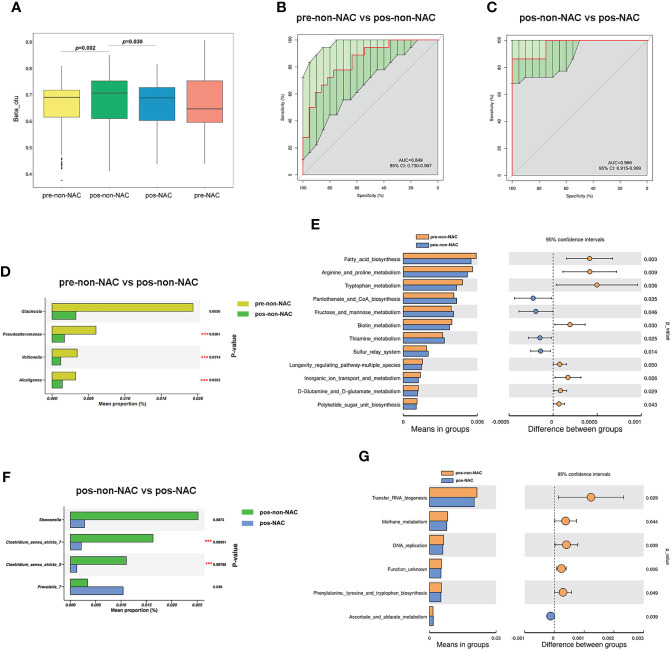
The effect of menopausal status on bacterial diversity between the non-neoadjuvant chemotherapy (non-NAC) and NAC groups. **(A)** The differences in beta diversity between the non-NAC and NAC patients with the status of premenopausal (pre-non-NAC and pre-NAC) and postmenopausal (pos-non-NAC and pos-NAC) calculated by the Wilcoxon signed-rank test. **(B, C)** The receiver operating characteristic (ROC) curve at the level of genus. **(D)** The microbial differences at the genus level between pre-non-NAC and pos-non-NAC groups. **(E)** The differences in predicted functional compositions of the breast normal adipose tissue (NAT) microbiome between pre-non-NAC and pos-non-NAC groups based on the Kyoto Encyclopedia of Genes and Genomes (KEGG). **(F)** The microbial differences at the genus level between pos-non-NAC and pos-NAC patients. **(G)** The differences in predicted functional compositions of the NAT microbiome between pos-non-NAC and pos-NAC groups based on KEGG by Tax4fun (***P-value < 0.05).

Beta diversity was distinctly different (*p* = 0.002) between premenopausal non-NAC (pre-non-NAC) and postmenopausal non-NAC (pos-non-NAC) patients as shown in **Figure 6A**. The AUC was 0.849, and the differences in microbiome composition between pre-non-NAC and pos-non-NAC groups could be explained at the genus level (**Figure 6B**). In the non-NAC patients, the genera of *Pseudoalteromonas*, *Veillonella*, and *Alcaligenes* were more enriched in the premenopausal women than the postmenopausal group (**Figure 6D**). The differential categories based on the KEGG level 3 between the pre-non-NAC and pos-non-NAC groups were majority related to metabolism (7/12, 58.33%). The “*fructose and mannose metabolism*” and “*thiamine metabolism*” were significantly increased in the pos-non-NAC group. Meanwhile, five categories, especially those related to amino acid metabolism (“*Arginine and proline metabolism*”, “*Tryptophan metabolism*”, and “*D-glutamine and D-glutamate metabolism*”) were both decreased in the pos-non-NAC patients (**Figure 6E**), indicating that the menopausal status could have an effect on the metabolic process of amino acids in the non-NAC patients.

The comparison of NATs from pos-non-NAC and postmenopausal NAC (pos-NAC) groups showed different microbiota compositions (*p* = 0.030) as shown in **Figure 6A**. The AUC between pos-non-NAC and pos-NAC groups was 0.966 (**Figure 6C**). In postmenopausal patients, the abundance level of *Clostridium_sensu_stricto_7* and *Clostridium_sensu_stricto_2* genera decreased in the NAC group compared with the non-NAC women (**Figure 6F**). Importantly, “*Methane metabolism*” decreased, and “*Ascorbate and aldarate metabolism*” increased in the pos-NAC group compared with the pos-non-NAC patients (**Figure 6G**).

## Discussion

The human microbiota plays an important role in host metabolism, digestion, and immunity. Disturbances in the microbiota can participate in the development of a variety of pathological conditions including cancer (26–28). BC has a richer and more diverse microbiome when compared with other tumors (9). This case-control study makes comprehensive comparisons of breast NAT microbiota between patients with BC and controls and demonstrates that a significant difference in the composition of bacteria exists between the two groups, which can be explained at the genus level. In addition, further analysis shows that the composition of genera in NATs of patients with BC significantly differed between the NAC and non-NAC groups. Moreover, the implication of NAC on the NATs microbial of patients with BC varied by menopausal status.

The relative abundance of the taxonomic composition of the NAT microbiome shows that *Proteobacteria* is the most abundant phylum, followed by *Firmicutes* and *Actinobacteria*, which is consistent with the results from previous literature (9, 29, 30). Interestingly, we also found the *Staphylococcus* was associated with BC metastasis (18) and was more bounteous in patients with BC than that in controls. Moreover, the correlation between *Staphylococcus* and cancer has been found at other body sites. A cohort study conducted by Gotland et al. reported that the genus *Staphylococcus aureus* bacteremia caused an increase in the risk of incident primary cancers, such as multiple myeloma, leukemia, sarcoma, liver, pancreatic, and urinary tract cancers (31). Sheweita et al. reported an association between a urinary tract infection with *Staphylococcus aureus* and bladder cancer (32). In addition, Staphylococcal superantigens can induce the disturbance of the immune environment and upregulate the expression of CD25, FOXP3, and IL-17 and play a role in the pathogenesis of cutaneous lymphomas (33). In this study, we observed that the genus *Burkholderia-Caballeronia-Paraburkholderia*, which is positively associated with body mass index (BMI) (34), enriches in BC group. Obesity is well known as a typical risk factor for BC (35, 36). The abundance level of genus *Shewanella* in patients with BC is higher than controls. The study conducted by Zhu et al. also showed that *Shewanella putrefaciens* is positively associated with estradiol levels (37).

We explored the associations between specific breast microbial taxa and breast tumor characteristics such as histologic grade and hormone receptor status. Consistent with the previous findings (38), we observed that the level of genera in phylum *Firmicutes* (*Clostridium_sensu_stricto_7*, *Clostridium_sensu_stricto_2*, and *Paenibacillus*) had mostly decreased in HER2-negative tumors, especially *f_Clostridiaceae_g_Clostridium*. However, upon reviewing the previous study conducted by Tzeng et al. (29) linking the breast microbiome and BC, some of our findings appear to be somewhat different. Contrary to the results discovered by Tzeng et al., we observed that the abundance levels of differential genera were more enriched in the ER-positive and non-TNBC groups. In addition, our study is the first to report that the genera *Vibrio*, *Pseudoalteromonas*, *RB41*, and *Photobacterium* are negatively correlated with the histological grade of BC.

In recent years, the correlation between microbiota and anticancer drugs is drawing a growing interest (39). Many chemotherapy agents change the composition of the microbiome, whereas microorganisms may also influence cancer progression by modulating the efficacy and the toxicity of drugs (40–43). As a common treatment of cancer, the effect of NAC on the diversity of microbiota in patients with BC is mainly focused on the intestinal flora. The role of NAC on breast tissue bacteria is unclear. A study conducted by Chiba et al. (18) indicates that the NAC could lead to a reduction in the microbiota diversity of breast tumors and cause a further increase in the abundance of *Pseudomonas* and a significant decrease in *Prevotella* level. Our study reveals that NAC can change the microbial composition of NAT in patients with BC and lead to a significant reduction in the abundance level of differential bacteria, especially the pathogenic bacteria, such as *Escherichia-Shigella*, *Clostridium_sensu_stricto_7*, and *Clostridium_sensu_stricto_2*. Of note, menopausal status may be associated with the NAC efficacy. When premenopausal women receive NAC, the adverse effects may include chemotherapy-related amenorrhea, infertility, and premature ovarian insufficiency (44, 45). In postmenopausal patients with BC, NAC can produce a reduction in tumor size and allow the use of more breast conservative therapy. Our study is the first to explore the impact of menopausal status on NAC therapeutic efficacy from the perspective of microbiota, and we found that NAC may associated with the microbiota community change of postmenopausal patients with BC (*p* = 0.030). In addition, PCoA analysis according to cancer subtypes (**Supplemental Figure 2**) further revealed that the bacterial structure of NATs and response to NAC differs between several BC subtypes. These findings are preliminary, and it is premature to propose a cause-and-effect relationship before more work is done. Our findings warrant attention in the larger sample size studies containing different cancer subtypes to elucidate the role of breast microbiota in the therapeutic outcome of BC and to explore the novel bacterial biomarkers that can predict the effect of treatment.

Notably, to further understand the potential mechanisms by which the breast microbiota drive host pathophysiology, we used a Tax4Fun prediction to analyze the different functional compositions of the NATs microbiome and found that the metabolism is more active in BC groups and that metabolic process may be involved in the BC-microbe interaction. Tumorigenesis is related to the reprogramming of cellular metabolism. Cancer cell metabolism can provide necessary nutrients from a frequently nutrient-poor environment and maintain cell viability (46). As described in a study by Giallourou et al. (47), the lipid profiles were higher in breast tumor-adjacent normal than healthy normal tissues, a finding that was attributed to the bacterial variation in the tissues. The activation of lipid and fatty acid pathways is most likely to be used as sources of energy that promotes BC cell growth in addition to pathogenesis. The previous investigation on PM_2.5_ and metabolism revealed that the impact of PM_2.5_ on the genera of the gut microbiota could partially mediate the relationship between PM_2.5_ and sphingolipid metabolism (48). In addition, the gut microbiota can affect host health by plasma metabolites (49). Metabolomics and microbiomics are both powerful approaches for identifying biomarkers. To identify novel mechanisms of BC and screen biomarkers, multi-omics analysis is particularly worthy of attention in future studies.

Our study has some specific strengths. First, a substantial literature gap still separates clinical observations and clinical interventions targeted at microbiota in BC. The findings of this study can provide data support for the association between breast microbiome and chemotherapy. Second, our results encourage further exploration of the establishment of the breast microbiome communication between breast microbes and metabolism and highlight the clinical implication of multi-omics analysis in screening the highly sensitive and specific biomarkers. Several limitations should be noted in this study. First, the conclusions of this study are preliminary for the limited sample size. Second, our results are limited by the inability to determine causality just as in other case-control microbiome studies. Further epidemiological studies with larger sample size and well-designed animal experiments are required to elucidate the role of breast microbiota in the therapeutic outcome of BC and to examine whether these associations are causal.

## Conclusions

Overall, we provide evidence supporting the conclusion that patients with BC differ from controls in breast NAT microbial composition and delineate specific breast bacterial profiles associated with tumor characteristics. Our results offer new insight into the relationship between NAC and breast microbiota and help to better characterize the breast microbial dysbiosis that occurs in patients with BC. Functional prediction findings may set the stage for multi-omics analysis to identify BC biomarkers in the future.

## Data availability statement

The data presented in the study are deposited in the NCBI BioProject repository, accession number PRJNA842933.

## Ethics statement

The studies involving human participants were reviewed and approved by Ethics Board of the Tianjin Medical University Cancer Institute and Hospital. The patients/participants provided their written informed consent to participate in this study.

## Author contributions

X-CC and N-JT conceptualized the study. AZ, YL, ZX, YY, ZY, XC, and XW contributed to data curation, investigation, and methodology. MY and JP verified the data. XL and XS wrote, reviewed, and edited the manuscript draft. All authors read and approved of the final manuscript.

## Funding

This work was supported by the grants from National Natural Science Foundation of China (nos. 81573115/H2601 and 81973002/H2601).

## Acknowledgments

We wish to thank the participants who volunteered to participate in the study.

## Conflict of interest

The authors declare that the research was conducted in the absence of any commercial or financial relationships that could be construed as a potential conflict of interest.

## Publisher’s note

All claims expressed in this article are solely those of the authors and do not necessarily represent those of their affiliated organizations, or those of the publisher, the editors and the reviewers. Any product that may be evaluated in this article, or claim that may be made by its manufacturer, is not guaranteed or endorsed by the publisher.
