# Supplementary material for: Breast microbiome associations with breast tumor characteristics and neoadjuvant chemotherapy: A case-control study
Source: Front Oncol. 2022 Sep 12;12:926920. doi: 10.3389/fonc.2022.926920 (PMC9510588; doi:10.3389/fonc.2022.926920)
Supplement: Supplementary file 1 [file DataSheet_1.docx]

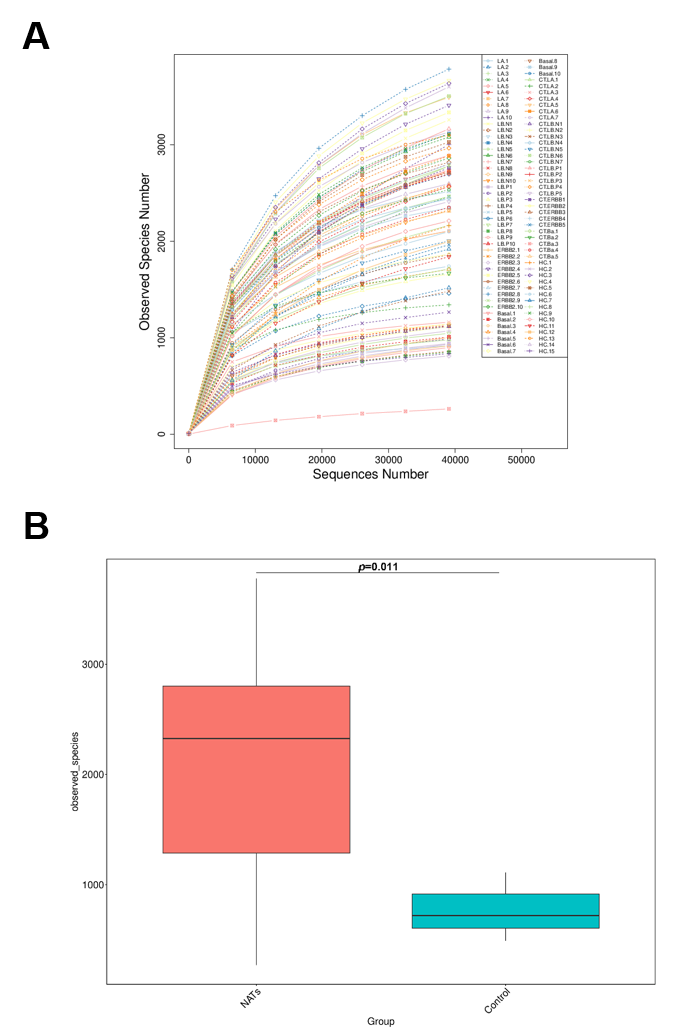


**Supplemental Figure 1.** Comparison of bacterial diversity between the breast normal adipose tissues (NATs) and cell lines. **(A)** The sample rarefaction curves of observed species numbers. The numbers of OTUs tend to plateau indicating that the amount of sequencing data is reasonable. (**B)** The alpha diversity was evaluated by the index of Observed Species indicating that the significant differences existed between the NATs and cell lines (MCF7, MDA-MB-231, and MCF-10A).


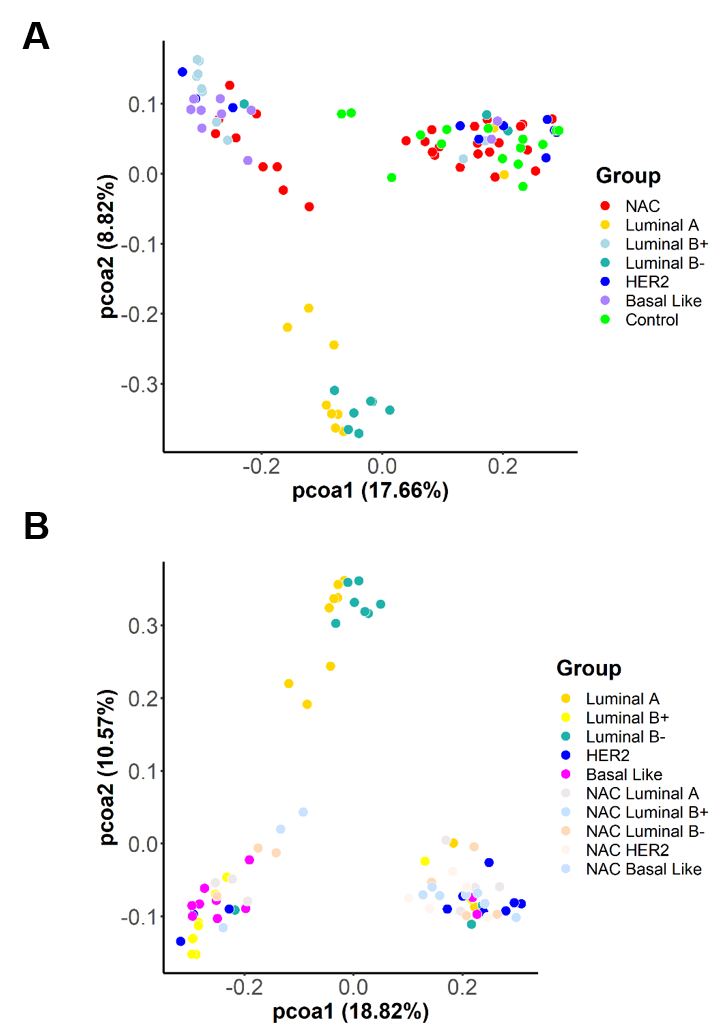


**Supplemental Figure 2.** Principal coordinate analysis (PCoA) of unweighted UniFrac distances. **(A)** The plot of PCoA between the different subtypes breast cancer patients and controls. (**B)** The PCoA plot of different subtypes of breast cancer between the neoadjuvant chemotherapy (NAC) patients and non-NAC patients.

**Supplemental Table 1.** The Spearman's correlation between breast bacterial profiles and breast tumor Nottingham grade.

| **Genera** | **r** | ***P*-value** |
| --- | --- | --- |
| *Vibrio* | -0.236 | 0.042* |
| *Pseudoalteromonas* | -0.297 | 0.010* |
| *RB41* | -0.376 | 0.001* |
| *Photobacterium* | -0.344 | 0.002* |
| *Streptococcus* | 0.243 | 0.035* |

**p* < 0.05
